# Awareness of electronic cigarettes in India: Findings from the 2016–2017 Global Adult Tobacco Survey (GATS)

**DOI:** 10.18332/tpc/156446

**Published:** 2023-01-25

**Authors:** Nidhi Jaswal, Garima Bhatt, Sonu Goel

**Affiliations:** 1 Department of Community Medicine and School of Public Health, Postgraduate Institute of Medical Education and Research, Chandigarh, India; 2 School of Medicine, University of Limerick, Limerick, Ireland; 3 Faculty of Human and Health Sciences, Swansea University, Swansea, United Kingdom

**Keywords:** India, GATS, ENDS, awareness, e-cigarettes, PECA

## Abstract

**INTRODUCTION:**

Electronic nicotine delivery systems (ENDS) have recently emerged as a public health threat globally. Despite the low proportion of e-cigarette users (1.22%) reported in the Global Adult Tobacco Survey-2, the Government of India enacted the Prohibition of E-cigarettes Act 2019 (PECA), prohibiting all forms of ENDS/ENNDS. The current analysis presents nationally representative findings on the level of awareness of e-cigarettes in India and its correlates and characteristics of those aware of e-cigarettes.

**METHODS:**

The current secondary analysis from GATS-2 among adults aged ≥15 years from all states and Union Territories of India used a standard protocol for data collection and management. A multi-stage cluster sampling design was used. The respondents who were aware of e-cigarettes were included (n=2524). Binomial logistic regression analysis was conducted, and adjusted odds ratios (AORs) with 95% CI, were calculated to measure the associations between independent and dependent variables.

**RESULTS:**

Only 3.4% of the respondents were aware (either heard or seen) of e-cigarettes and their awareness was found significantly higher among males (AOR=2.07; 95% CI: 1.90–2.24), urban population (AOR=2.83; 95% CI: 2.61–3.07), and higher education (AOR=0.41; 95% CI: 0.38–0.45).

**CONCLUSIONS:**

Public awareness campaigns about the harms of e-cigarettes and the law (PECA) need to be rolled out in urban and rural areas. Capacity-building exercises of implementers and enforcers at the grassroots level could also support communicating the harms to hard-to-reach groups. Further, regular compliance monitoring of the legislation and prosecution of violators would facilitate its effective implementation at the national and sub-national levels.

## INTRODUCTION

With about 267 million tobacco smokers, India is the world’s second-largest consumer of tobacco products. There are 100 million tobacco smokers among them, and over 199 million people who use smokeless tobacco^1^. Recent global trends have shown the emergence of electronic nicotine delivery systems (ENDS; also known as electronic cigarettes or e-cigarettes). These non-combustible tobacco products, also known by many names such as vapes, e-hookahs, vape pens, e-cigars, and e-pipes, are marketed as tobacco-free nicotine delivery battery-operated devices which produce an aerosol by heating a solution containing nicotine, among other things, instead of burning tobacco leaves in traditional cigarettes^2^.

Numerous studies have highlighted an increased awareness about e-cigarettes in developed countries^3,4^. Little is known about e-cigarettes in middle-income countries, many of which, like Mexico, banned sales and marketing of e-cigarettes^5^. Understanding country-specific data on e-cigarettes is vital in understanding their growth patterns so that appropriate public health interventions and policies can be planned. To address the need for nationally representative surveillance data on e-cigarette use, the Global Tobacco Surveillance System of a few countries, including Indonesia, Malaysia, Qatar, and Greece, incorporated questions on e-cigarettes into the Global Adult Tobacco Survey (GATS) in 2011^6^. However, India incorporated these questions in the second round of GATS (2015–2016)^1^. The GATS (2015–2016) was carried out before the promulgation of the Act. Despite the low proportion of e-cigarette users (1.22%) reported in the survey^1^, the Government of India went ahead with placing a ban on the same. The current analysis presents nationally representative findings on the level of awareness of e-cigarettes in India and its correlates.

## METHODS

### Data source

This was a secondary data analysis from Global Adult Tobacco Survey 2 (GATS 2), a nationally representative household survey conducted in 2015–2016 of people aged ≥15 years. A standard protocol included a questionnaire, sample size and design, data collection, and management procedures^1^.

### Sample size and design

The analysis is based on a total of 74037 completed interviews (33772 men and 40265 women). A multi-stage, geographically clustered sample design was used. One individual from each chosen household was selected randomly for participation^1^. To identify sociodemographic factors affecting awareness of e-cigarettes, an analysis was run on all the participants (n=74037). For analyzing the use of e-cigarettes, the people who were aware of e-cigarettes were included (n=2524). For calculating the wealth index, question A06 was considered. A composite score of each respondent was calculated, which was further categorized into five quintiles: 0–20% 1st, 20–40% second, 40–60% third, 60–80% fourth, and 80–100% fifth.

### Operational definitions as per GATS (2016–2017)^1^

1. Awareness about e-cigarettes: ‘Have you ever heard or seen e-cigarettes?’ (EC1)
2. Current use of e-cigarettes: ‘Do you currently use e-cigarettes on a daily basis, less than daily, or not at all?’ (EC2)
3. Ever use of e-cigarettes: ‘Have you ever, even once used e-cigarette?’ (EC3)
4. Reason to use e-cigarettes: ‘What is the main reason why you use electronic cigarettes? (EC4)

The independent variables extracted for the current study were:

1. Sociodemographic variables: sex, age, education level, residence, occupation, and wealth index (AO1, AO3, AO4, AO5, AO6)
2. Current tobacco smokers/non-smokers: ‘Do you currently smoke tobacco on a daily basis, less than daily, or not at all?’ (B04 to B07)
3. Former smokers/never-smokers: ‘In the past, have you smoked tobacco on a daily basis, less than daily, or not at all?’ (B11)
4. Attempted to quit smoking: ‘Which of the following best describe your thinking about quitting smoking?’ (DO1)
5. Noticed health warnings on cigarette packages: ‘In the past 30 days, did you notice any health warnings on cigarette packages?’ (GO2)

### Statistical analysis

Analysis was done using SPSS software version 16.0. The awareness about e-cigarettes and 95% confidence intervals were calculated. Binomial logistic regression analysis was conducted and adjusted odds ratios (AORs) with 95% CI, were calculated to measure the associations between sociodemographic factors, current or former tobacco smoking, attempt of quitting smoking, exposure to health warnings on cigarette packs, and e-cigarette awareness/use. Gender, age, education level, residence, occupation, wealth index, and current tobacco user, were the factors that were adjusted for.

## RESULTS

### Awareness of e-cigarettes

A total of 2524 (3.4%) respondents were aware of e-cigarettes (either heard or seen). Nearly two-thirds of them were males (62.7%), over half were from the age group of 25–44 years (55%), urban residents (60.3%), and employed (57%). The awareness levels significantly reduced with increasing age. Besides, the awareness was 2.83 times (AOR=2.83; 95% CI: 2.61–3.07) higher in the urban population than in the rural. Those who were financially sound or outgoers such as students had higher odds of awareness about e-cigarettes (AOR=2.78; 95% CI: 1.02–1.55). The respondents who noticed health warnings on cigarette packages were more aware of the e-cigarettes (AOR=2.41; 95% CI: 2.20–2.63). The awareness of e-cigarettes was less among the respondents belonging to the lowest wealth indices (OR<1) which was statistically significant (Table 1).

**Table 1:** Awareness of e-cigarettes among adults aged ≥15 years in India, GATS 2016–2017 (N=2524)

*Characteristics*	*Awareness of e-cigarettes^a^*	
	*n (%)*	*AOR^c^ (95% CI)*
**Gender**		
Male	1587 (62.9)	2.07 (1.90–2.24)***
Female (Ref.)	937 (37.1)	1
**Age** (years) (n=2418)^a^		
15–24	529 (21.9)	3.22 (2.55–4.06)***
25–44	1330 (55.0)	2.80 (2.24–3.50)***
45–64	475 (19.6)	1.81 (1.44–2.29)***
≥65^b^ (Ref.)	84 (3.5)	1
**Residence**		
Urban	1522 (60.3)	2.83 (2.61–3.07)***
Rural (Ref.)	1002 (39.7)	1
**Education level**		
No formal education/less than primary	206 (8.2)	0.06 (0.05–0.07)***
Primary/less than secondary	453 (17.9)	0.18 (0.16–0.20)***
Secondary/high secondary school	901 (35.7)	0.41 (0.38–0.45)***
College/university or higher^b^ (Ref.)	964 (38.2)	1
**Occupation**		
Government employee	339 (13.4)	4.46 (3.59–5.53)***
Non-government employee	493 (19.5)	3.40 (2.76–4.17)***
Daily wager	181 (7.2)	0.53 (0.41–0.67)***
Self-employed	429 (17.0)	1.26 (1.02–1.55)*
Student	402 (15.9)	2.78 (1.02–1.55)***
Homemaker	563 (22.3)	0.89 (0.72–1.08)
Retired/unemployed^b^ (Ref.)	116 (4.6)	1
**Wealth index**		
Lowest	290 (11.5)	0.08 (0.07–0.09)***
Second	433 (17.2)	0.15 (0.14–0.17)***
Middle	293 (11.6)	0.28 (0.24–0.32)***
Fourth	591 (23.5)	0.45 (0.41–0.51)***
Highest (Ref.)	913 (36.2)	1
**Current tobacco smokers**		
Yes	420 (16.6)	0.73 (0.66–0.81) ***
No (Ref.)	2104 (83.4)	1
**Current e-cigarette users**		
Yes	31 (1.2)	0.84 (0.67–1.29)
No (Ref.)	2493 (98.8)	1
**Noticed health warnings**		
Yes	1820 (72.1)	2.41 (2.20–2.63))**
No (Ref.)	704 (27.9)	1
**Attempted quitting smoking** (n=420)^a^		
Yes	169 (40.2)	1.34 (1.01–1.63)
No (Ref.)	251 (59.8)	1

a Difference in sample is due to the missing values in the data set.

b Constant value.

c AOR: adjusted odds ratio, adjusted for gender, age, education level, residence, occupation, wealth index, and current tobacco user.

* p<0.05,

** p<0.01,

*** p<0.001.

## DISCUSSION

The current study highlighted a low level of awareness about e-cigarettes among the adult population in India. This is probably the first large study from India that documents the awareness of e-cigarettes and the characteristics of vapers before the ENDS ban in the country. Despite a lower awareness and use, the promulgation of the ENDS ban in the year 2019 demonstrates the strong commitment of the Government of India to counter tobacco industryled nefarious strategies to promote e-cigarette use. The Indian e-cigarette market reached a value of $7.8 million in 2018, and it is further predicted to witness a compound annual growth rate of 26.4% during the forecast period (2019–2024)^7^. Owing to the limited literature available for low- and middle-income countries, the current study was planned to develop an evidence base from India on the level of awareness of e-cigarettes.

Awareness of e-cigarettes was found to be more among young males in the current study. Peer pressure, social media exposure, and trend were major factors for its higher awareness in this age group^3,4^. The working population and students were found to be more aware of e-cigarettes than the unemployed, which may be due to their high likelihood of trying regular tobacco products and then gradually increasing intention to indulge in other smoking products, as has also been demonstrated in other studies^8^. E-cigarette marketing focusing on the productive age group could be an essential determinant in increased awareness in this group^9^. The widespread availability of articles explaining how to procure and use ENDS on the internet has also led to increased awareness among the youth^10^. Tobacco smokers were less aware of e-cigarettes compared to non-smokers. This may be due to the perception that e-cigarettes are less harmful than conventional tobacco smoking and perceived these to be a cessation tool^11^. Further, it also could be because at the time of survey, the respondents had less exposure to scientific evidence about the harm of e-cigarettes and relied on anecdotal evidence. Also, perhaps the smoker prefers to buy a single stick instead of the entire package and hence misses out on the pack warning.

The current study provides preliminary evidence on the characteristics of those aware of e-cigarettes and their factors before the ban, which could be helpful for policymakers in making apt decisions and designing appropriate policies for strict enforcement and implementation of the ENDS ban.

### Limitations

The cross-sectional nature of the survey inherently limits us from establishing any causal relationship and only represents the situation at a time. The sample size was not large enough to provide precise estimates. Since it was a household survey and the sample was randomly selected on pre-determined criteria, there was a possibility of missing the significant population of potential young users studying and working. Besides, weighted analysis was not possible due to missing data sets.

## CONCLUSIONS

Overall there was low awareness of e-cigarettes among the Indian population. Awareness was higher among males, younger age group, students, urban population, respondents who had a higher level of education, and the highest wealth quintile. Therefore, there is a need for explicitly targeted information and education campaigns (worksites and universities) about ENDS. Besides, sensitization and capacity building of stakeholder departments for effective implementation of the legislation [The Prohibition of Electronic Cigarettes (Production, Manufacture, Import, Export, Transport, Sale, Distribution, Storage and Advertisement) Act, 2019]^12^ and compliance monitoring should be carried out. A robust surveillance mechanism should monitor e-retail stores for sales and digital platforms regarding the advertisement of ENDS and like products.

## Data Availability

Data supporting this research are available from https://www.cdc.gov/tobacco/global/gtss/gtssdata/index.html.
